# Quality of Capacity Documentation in Older-Adult Psychiatry Inpatients in the Black Country

**DOI:** 10.1192/bjo.2026.11827

**Published:** 2026-06-30

**Authors:** Aparna Prasanna, Nandini Ajoy

**Affiliations:** 1 Black Country Healthcare NHS Foundation Trust, Wolverhampton, United Kingdom; 2 The Royal Wolverhampton NHS Trust, Wolverhampton, United Kingdom

## Abstract

**Aims:**

This audit aimed to evaluate the quality, completeness, and legal compliance of capacity assessment documentation for older adult psychiatry inpatients at an older adult inpatient ward in the Black Country. The standards were based on the Mental Capacity Act (MCA) 2005 and its Code of Practice, which require that capacity assessments are properly recorded, with clear rationale and evidence for each element of the functional test. The MCA emphasises supporting individuals in decision-making and ensuring that decisions made for those lacking capacity are in their best interests and represent the least restrictive option. Recent literature, such as Ngwenya (2023), highlights that high-quality documentation is essential for safeguarding patient autonomy and ensuring legal defensibility, yet audits often reveal significant variability in practice.

**Methods:**

The audit reviewed all MCA and Best Interests forms completed for inpatients admitted to the older adult inpatient ward between 1st March and 1st September 2025. Data were collected from the RiO electronic patient record system. Seven key standards were assessed: decision-specific documentation, support to decide, completion of the functional test (understand, retain, use & weigh, communicate), diagnostic link to mental impairment, documentation of best interests decisions, consultation with relevant others, and proper completion by the assessor. A total of 61 forms from 39 admissions were analysed using a pro forma with “Yes/No” ratings for each standard.

**Results:**

Compliance rates varied: decision-specific documentation (90%), support to decide (70%), functional test (60.6%), diagnostic link (84.6%), best interests (32.4%), consultation (29.7%), and completed by (100%). Only 14.7% of forms met all standards. Three patients lacked a capacity assessment on admission, and one form had no result recorded. Good documentation included detailed evidence of patient understanding and reasoning; poor examples were vague or lacked rationale. The most significant gaps were in documenting best interests decisions and consultation with family or advocates. These findings echo national trends, where audits frequently identify insufficient detail in functional assessments and limited evidence of patient-centred approaches (Ngwenya, 2023).

**Conclusion:**

The audit revealed substantial variability and gaps in capacity documentation. Recommendations include regular training on MCA documentation, peer review and supervision of assessments, appointing capacity champions on the ward, and establishing feedback loops through audit presentations at governance meetings. These measures, aligned with national best practice, will help ensure legal compliance, protect patient rights, and improve the quality of care for vulnerable older adults.

